# Parental reassurance concerning a feverish child: determinant factors in rural general practice

**DOI:** 10.1186/s12875-017-0686-1

**Published:** 2018-01-09

**Authors:** Anthony Chapron, Marc Brochard, Chloé Rousseau, Anne-Charlotte Rousseau, Martine Brujean, Laure Fiquet, Virginie Gandemer

**Affiliations:** 1 Univ Rennes, Department of General Practice, 2 av du Pr Léon Bernard, F-35043 Rennes, France; 2 Univ Rennes, CHU Rennes, Inserm, CIC 1414 [(Centre d’Investigation Clinique de Rennes)], F-35000 Rennes, France; 3 CHU, Département d’Hémato-oncologie Pédiatrique, Rennes, France

**Keywords:** Fever, Preschool child, General practice, Health knowledge, Attitudes, Practice*

## Abstract

**Background:**

Acute fever is the most common pediatric condition encountered in general practice and a source of parental concern that can result in inappropriate behavior. The main objective of this study was to describe and quantify parental reassurance concerning their feverish child in the context of visits to rural general practitioners (GPs).

**Method:**

The study included the parents of 202 feverish children, aged from 3 months to 6 years, consulting 13 representative rural GPs. Questionnaires were administered before and after the consultation. Uni- and multivariate analysis were performed to study variations of the levels of concern and associated factors.

**Results:**

The duration of fever was 1.3 days (± 1.1). The mean score for parental concern was 4.8 out of 10 (± 2.2) before, and 2.4 (± 1.9) after the consultation (*p* < 0.0001). The concern correlated with the timing of the appointment relative to the usual wait (*p* = 0.0002), and a lack of knowledge about fever complications (*p* = 0.013).

**Conclusion:**

Facilitating access to consultations with a GP within the expected timeframe reduces parental concern. Increasing parental education about fever is also necessary.

**Electronic supplementary material:**

The online version of this article (10.1186/s12875-017-0686-1) contains supplementary material, which is available to authorized users.

## Background

Acute fever in children is defined as an elevation of the core temperature above 38 °C in the absence of physical activity for a normally dressed child at ambient temperature [1]. It is a frequent cause of consultation, notably because it is a source of concern for the parents [2, 3]. This physiological reaction to infection is negatively perceived, even though a clinically isolated, well-tolerated acute fever does not require a medical consultation within the first 48 h after its occurrence. Parents may consider it to be a danger to their child or worry that it reveals a serious illness. Importantly, parental concern may lead to inappropriate behavior [2–5], which must be avoided. “Parental reassurance” is a parental behavior used to sooth the distress of their child during a pediatric procedure [6], but it can also denote the decrease of parental concern (reassurance of parents).

Parental concern is a subjective phenomenon and consulting a doctor may affect it. Moreau et al. showed that simply consulting a doctor has a beneficial effect on adult patients [7]. This is known as the “doctor effect”. This effect is measurable by objective clinical (ex. blood pressure, analgesic consummation) and biological criteria (ex. glycated hemoglobin) for diverse acute or chronic diseases. This concept has not been studied using subjective criteria. Parental concern about fever in their children has been studied in pediatric hospitals [8], ambulatory clinics [9], and schools [10]. However, in France, children are mostly followed by a general practitioner (GP): 5% of children under 2 years of age are followed solely by a pediatrician, 40% solely by a GP (exclusively in rural areas), and 55% by both [11].

Improving the health of people living in rural areas through access to effective healthcare is recognized to be an important issue because *“[rural] share a context of relative isolation from large population centers and major healthcare facilities and typically suffer health inequities and unmet healthcare needs”* [12].

Our study focused on parental reassurance in the context of parental concern for a child with acute fever while waiting for a consultation with a GP in rural areas. Our principal objective was to describe and quantify parental reassurance in the context of rural general practices. The secondary objective was to determine the factors associated with the reduction of parental concern.

## Methods

A cross-sectional study in rural general practices was performed to evaluate the immediate parental reassurance.

### GP investigators

The GP investigator offices were selected in rural areas, according to their definition in the medical demography atlas of the College of Doctors [13]. Rural areas had to be sufficiently distant from emergency care services and pediatric offices, and not receive home urgent care services, such as *“SOS Médecins”,* to investigate the use of nearby general practitioners. Eligibility of GPs was based upon a pediatric recruitment close to regional average (variance of less than 10%). This was determined using their individual activity and prescription records (*relevés individuels d’activité et de prescription* or RIAP, provided by GPs), indicating the percentage of consultations for children under 6 years. The offices were required to have a medical secretary and consultations planned by scheduling appointments. The GP investigators had to be representative of the GPs of rural zones of their *département* (a large administrative district) by age, sex, and their status as general practice university lecturers, based on their records [13]. All GPs of one *département* were screened. A GP had to meet all selection criteria and to consent to take part in this study to be included.

### Population

All children aged from 3 months to 6 years, brought for a consultation by at least one of their parents for acute fever (less than 5 days), were eligible. During a six-month period from August to January 2012, parent(s) with a feverish child were systematically invited by the medical secretary to participate in the study, either during the scheduling of the appointment or upon their arrival at the office. There were no exclusion criteria. If the parent(s) agreed, two anonymous questionnaires were given: one to be completed in the waiting room before and the other immediately after the consultation.

### Questionnaires

Questionnaires (Additional file 1) were developed for this study by three general practitioners and one pediatrician. The first questionnaire collected the characteristics of the child (age, sex, history, birth rank), the socio-demographic characteristics of the parents, and their knowledge about fever (open question). In addition, parents were asked to provide two additional pieces of information with respect to the timing of the consultation – i.e. the time between the onset of fever and the consultation - and to the amount of time that the parents would have expected between the onset of fever and the consultation. A self-administered quantitative tool measured parental concern, which is a subjective phenomenon. It was scored on a horizontal numerical visual analogue scale [14]*,* to allow an easy and rapid measure: 0 for no concern to 10 for maximal concern. The second questionnaire queried the level of concern (same scale from 0 to 10), and the reasons for the change (or not) of the concern level of the parent who was present (open questions). Questionnaires were pre-tested in other rural areas than those included in this study, and validated after minor revisions.

Since our goal was to assess the symptom “fever” regardless of the diagnosis, the level of concern was studied independently of the diagnosis of the GP at the end of the consultation. The parents could only participate once in the study during the data-collection period.

### Statistical analysis

We postulated that parental reassurance by consulting a doctor might be due to the “doctor effect”. Thus, the intended sample size was 200 children, based on published data from randomized controlled trials concerning the “doctor effect” [7, 15] and the definition of parental reassurance as a decrease in parental concern by at least 50% following the consultation.

Inclusion by medical secretaries was hence stopped when 200 children with before and after questionnaires were referenced.

Excel© 2008 and SAS© 9.2 software was used for data entry and statistical analyses. A descriptive analysis of the data was performed (mean, standard deviation, median). We compared means by the Student test, medians by the Wilcoxon test, and proportions by the chi-2 and Fisher tests. We verified the normal distribution of samples using the Kolmogorov-Smirnov test. We processed the open questions by thematic analysis of the lexical content and manual post-coding. We performed a consistency check between the responses to the open questions and the variation of the parental concern score before and after the consultation: in the case of non-adherence, the questionnaire was not included in the analysis. We regrouped the answers to the open questions on the medical antecedents of the children and the complications of fever according to the categories of the French version of the ICPC-2 [16]. We classified the knowledge of parents into four categories: adequate (all of the responses were correct), intermediate (at least one correct response), erroneous (no correct response), and no knowledge (no answer).

Multivariate analyses focused on the level of concern before consultation and the reduction of the concern score before and after, by adjusting the model for explanatory variables of the descriptive analysis. Two by two comparisons according to the Bonferroni correction were used for statistically significant variables of more than two levels.

The results for which *p* < 0.05 were considered to be significant.

### Ethics

The study received approval from the Ethics Committee of the Department of General Medicine of Rennes 1 University, competent for all involved rural areas. Questionnaires were provided after the parents were informed by medical secretary about the content of the study and its anonymous and non-obligatory nature. No payment was offered. So parents’ participation in the study was voluntary after verbal consent.

## Results

A total of 17 GPs was eligible for the study, of which 13 from four practices with medical secretaries participated as investigators. The practices were between 45 and 75 km far from the closest hospital with a pediatric unit and an average of 55 km far from the closest pediatrician in private practice. The pediatric activity of the 13 GPs reflected the regional pediatric activity (29.8% for their activity vs. 21.7% for the region). The 13 GP investigators were representative of the GPs of the district: mean age (53 years ±7.1), sex (69% males), and status as general practice university lecturers (3 of 13 GPs i.e. 23%).

The study included 202 consultations, spanning three seasons (summer, autumn, and winter).

### Population

The average age of the children included in the study was 30.3 months ±16.4. Of these, 65 (32.2%) were an only child (Table 1). Fifty children (24.8%) had medical antecedents, primarily respiratory, uro-digestive or otorhinolaryngologic.

**Table 1:** characteristics of the study population

Children n (%)	202; girls: 99 (49); boys: 103 (51)
Age (months), mean, standard deviation	30.3 ± 16.4
Number of siblings	
• 1	65 (32.2)
• 2	89 (44.1)
• 3	36 (17.8)
• ≥ 4	11 (6.0)
Presence of one or more antecedents^a^	50 (24.8)^c^
• respiratory	22 (35.5)
• uro-digestive	16 (25.8)
• otorhinolaryngology	11 (17.7)
• others	11 (17.7)
• neurological	2 (3.2)
Parents n (%)	
Accompanying parent is the mother	158 (78.0)
Two parent family	192 (95.0)
Socio-professional category of the accompanying parent^b^	
• farmer	0 (0)
• artisan, merchant, business leader	13 (6.6)
• manager, intellectuel profession	11 (5.6)
• white collar worker	50 (25.4)
• employee	62 (31.5)
• laborer	18 (9.1)
• retired	0 (0)
• no employment	43 (21.8)
Parental knowledge about fever:	
• definition of fever (*n* = 202)	
○ < 38 °C	23 (11.4)
○ 38 °C	116 (57.4)
○]38 °C-38°5C]	50 (24.8)
○ > 38°5C	13 (6.4)
• Fever is dangerous? (*n* = 202)	yes: 186 (92.1)
• Cited complications^a^ (*n* = 186)	
○ neurological	98 (47.6)
▪ including convulsion	33.8%
○ metabolic	42 (20.4)
▪ including dehydration	15.4%
○ others	46 (22.3)
Sources of information of the parents (*n* = 202) (multi-choice questions)	
○ general practitioner	156 (77.2)
○ experience	139 (68.8)
○ entourage	83 (41.1)
○ child’s health record^d^	38 (18.8)
○ pediatrician	33 (16.3)
○ pharmacist	19 (9.4)
○ media	11 (5.4)

^a^classification according to the devices or systems of the ICPC-2 (French version)

^b^socio-professional category of the parent accompanying the child during the consultation (INSEE categories)

^c^50 children have antecedents, concerning 62 pathologies cited by their parents

^d^“carnet de santé” in France: booklet issued at birth that contains the medical history of the child

The accompanying parent was primarily an employee (62 parents; 31.5%) or engaged in a white-collar profession (50; 25.4%).

Children had fever for 1.3 days (± 1.1) prior to the consultation. Parents said they had waited 1.2 days (± 0.8) before visiting a doctor. Most (87.6% 177 parents) stated that they had measured the temperature of their child before the visit. Their knowledge about their child’s fever was essentially based on the information provided by the GP, their personal experience, or the advice of their entourage. For 92.1% of parents, the fever represented a danger; two-thirds considering 40¨C as a dangerous threshold. Convulsions (33.8%) and dehydration (15.4%) were the most frequently cited complications among those listed by parents.

### Parental reassurance

The average level of parental concern as measured by the concern score (CS) was 4.8 out of 10 (± 2.2) before the consultation and 2.4 (± 1.9) after (*p* < 0.0001). The variation of the CS, i.e. the change in the level of parental concern, was not related to the practice. The concern score decreased from 1.9 to 2.7 points between the four practices (*p* = 0.79). Immediately after the consultation, 169 parents (83.7%) stated that they had been reassured, 14 (6.9%) that they were still as concerned as before, and two (1.0%) that they were more concerned. The most reassuring element of the consultation was the communication of a diagnosis (Table 2). The lack of knowledge of the parents concerning fever complications was associated with a higher level of concern before the consultation (*p* = 0.013) and an absence of parental reassurance (reduction of the concern score < 50%, *p* = 0.049; Table 3).

**Table 2:** Effect of the consultation on parental concern (*n* = 202)

Parents declaring that they are reassured, n (%) *After checking for consistency with the evolution of the CS*	169 (83.7) *152 (91.6)*
• by communicating a diagnosis	110 (72.4)
• by examining the child	67 (44.1)
• by questioning and listening to the parents	30 (19.7)
• by giving a prescription	10 (6.6)
• other	6 (3.9)
Parents declaring as or more concerned, n (%) *After checking for consistency with the evolution of the CS*	16 (7.9) *14 (8.4)*
• waiting for a diagnosis	8 (57.1)
• discussion with doctor not reassuring	5 (35.7)
• waiting for treatment	1 (7.1)
• because of the diagnosis	1 (7.1)
Undecided, n (%)	17 (8.4)

**Table 3:** Parameters influencing parental concern and the change in the level of parental concern (univariate analysis)

Parameters	parental concern before consultation		change in the level of parental concern		
	concern score (CS) (mean, standard deviation)	*p*	Reduction of concern score (CS)		*p*
			> 50% subjects n (%)	< 50% subjects n (%)	
Medical antecedents of the child		0.215			0.990
• yes	5.12 ± 2.1		25 (31.6)	20 (31.7)	
• no	4.63 ± 2.2		54 (68.4)	43 (68.3)	
Only child		0.159			0.379
• yes	5.08 ± 2.3		31 (30.1)	29 (36.3)	
• no	4.61 ± 2.1		72 (69.9)	51 (63.8)	
Socio-professional category of the accompanying parent		0.435			0.222
• artisan, merchant, business leader	4.96 ± 2.1		3 (2.9)	8 (10.5)	
• manager, intellectual profession	5.41 ± 2.2		5 (4.9)	6 (7.9)	
• white collar worker	4.39 ± 2.2		28 (27.5)	18 (23.7)	
• employee	4.66 ± 2.3		29 (28.4)	25 (32.9)	
• laborer	5.50 ± 2.3		10 (9.8)	6 (7.9)	
• no employment	4.86 ± 1.9		27 (26.5)	13 (17.1)	
Complications of fever: parental knowledge		*0.013*			*0.049*
• adequate	4.50 ± 2.1		44 (42.7)	27 (33.8)	
• intermediate (at least one correct response)	4.77 ± 2.3		18 (17.5)	8 (10.0)	
• erroneous (no correct response)	5.98 ± 1.9		9 (8.7)	17 (21.3)	
• no knowledge	4.56 ± 2.1		32 (31.1)	28 (35.0)	
The difference between the actual consultation date and that preferred by the parents		*0.0002*			0.896
• more than 0.5 days before	3.45 ± 2.2		10 (12.7)	8 (11.8)	
• between 0.5 days before and 0.5 days after	4.83 ± 2.1		54 (68.4)	45 (66.2)	
• more than 0.5 days after	5.77 ± 1.8		15 (19.0)	15 (22.1)	

The level of parental concern before the consultation was directly proportional to the difference between the actual timing of the consultation and that preferred by the parents (correlation coefficient = 0.29, *p* = 0.0002): the longer the wait the greater the concern. There was no significant association between the level of concern before the consultation or the change of the level of concern due to the consultation, and the characteristics of the children or the parents (Table 3).

Multivariate analysis identified only the timing of the appointment was associated with the average level of parental concern before the consultation (*p* = 0.013). After adjusting the level of concern for the knowledge of the parents, the communication of the diagnosis, and the age of the child (Bonferroni correction), there was a significant difference (*p* = 0.0096) in the level of concern before consultation between parents who obtained an appointment at least half-a-day earlier than their normal wait (adjusted mean CS of 3.9/10) and those who had to wait half-a-day more than usual (adjusted mean CS of 5.9/10). However, the wait for the appointment was not significantly associated with the reduction of at least 50% of the CS after the consultation (OR 1.61 [0.81; 3.20]).

## Discussion

The lack of parental knowledge about fever and timeframe of the appointment (half-a-day more or less than their usual waiting time) were identified as factors associated with the level of concern before the GP consultation. A more than 50% reduction in the level of concern was associated in univariate analysis with parental knowledge about fever.

Acute fever is the most common pediatric condition encountered in general practice. The present study allowed an objective analysis of the subjective phenomenon of parental reassurance by describing and quantifying the reduction of their concern immediately after the consultation in the context of pediatrics and primary care, which was not previously done.

We chose not to study babies of less than 3 months, because of the specific character of fever in children under this age [17], nor children of more than 6 years of age, to obtain a homogeneous cohort of children and better select GP investigators (*RIAP* criteria). We also wanted to be able to compare our study with other French studies on parental perceptions and knowledge about their children’s fever [18–20].

Parental concern about a child’s fever has been described previously [2, 3], but never measured. We report that medical consultation had a positive effect (*p* < 0.0001) as determined using a threshold of a 50% reduction in the level of concern, or concern score. This effect is all the more remarkable because the preexisting level of concern, prior to scheduling the doctor’s appointment, was possibly even higher, although it was not evaluated in our study.

There was no significant difference in parental concern levels between socio-professional categories of the parents. The literature is divergent on this point. Some authors describe a significant link between the anxiety concerning the child’s fever and the level of education of the parents [2, 3, 11, 21], whereas others have shown that their socio-professional level influenced the attitudes of the parents [19]. Our study differed from previous studies because our population was entirely rural and mostly followed by a GP. This suggests that the proximity, rapidity, and frequency of contact with the family doctor is associated with the absence of differences in the perception of the parents of different socio-economic status. For this study, only the socio-professional category of the accompanying parent was taken into consideration since it was only possible to quantify the concern of that parent about their child’s fever.

### Increasing parental education about fever is necessary

Parents worried even if they had appropriate knowledge. There was, however, a significant association between parental concern and their lack of knowledge concerning the complications of fever (*p* = 0.013). It is therefore important that doctors educate parents about the physiology of fever symptoms and their management. Educating parents is part of the role of GPs [22], but educative tools [23, 24] designed in conjunction with pediatricians [25] and public health strategies can also contribute. Parental concern is essentially linked to the idea that fever is synonymous with danger for the child, resulting in complications of which the parents have incorrect or inadequate knowledge. It would be valuable to reinforce educational campaigns directed to the general population [26], such as the information in the child’s health record *(carnet de santé)* on what to do in case of fever in a child older than 3 months.

### Availability of GP is crucial

We have shown that this concern is real, and leads to the scheduling of an appointment to see the GP as soon as possible. The lag between the appointment time preferred by the parents and the actual consultation was the principal factor that was associated with parental concern before the consultation. The parents of this study stated that they would have preferred a delay of 1.2 days before the consultation for their child’s fever, whereas the consultation was, on average, 1.3 days after its apparition. This timing is consistent with other French studies [17, 19]. We found that the greater the difference between the habitual wait before the consultation and the actual wait, the greater the parental concern. Keeping parents waiting also keeps them in a state of concern, and could lead to inappropriate behavior.

### Reassurance as a *“doctor effect”*

Consulting the doctor is a source of parental reassurance, indicating that the concept of the “doctor effect” could influence the subjective phenomenon of parental concern. The originality of our study is the assessment of the “doctor effect” on the person accompanying the patient, rather than on the patient, as usually studied in primary care [8]. Parents play a fundamental role in pediatric issues, necessitating the study of parental concern and parental reassurance. The key element reported by parents that led to their reassurance was the announcement of the diagnosis. The attention given to the clinical examination of the child and listening to the parents is also important for their reassurance. The average parental concern score decreased due to the doctor-parent interaction during the examination of feverish child. It is therefore also essential to remind GPs of their ability to reassure parents: medication is not the main issue of these frequent consultations. These findings provide further evidence for the “doctor effect”, defined as a cognitive-emotional element of the consultation [7]. The “doctor effect” is, indeed, an integral element of the management of fever. Parental concern is little reduced by simply providing a prescription, but rather is diminished by the doctor’s availability and the quality of the dialogue with the doctor. Also, the GP is the major source of information about fever for parents in rural areas; in urban zones, the role of the pediatrician may be more important [10].

### Study limitations

The present study was a cross-sectional and not a longitudinal study. Its aim was to evaluate the *immediate* effect of reassurance and not to follow-up patients, which should be considered in forthcoming studies.

A numerical scale was chosen to measure the evolution of the concern level before and after the consultation with the GP. We were solely interested in the change in concern level to quantify the beneficial effect of the consultation on parental reassurance. We did not study parental anxiety, in the medical sense, and therefore did not use a validated anxiety scale. A simple numerical scale from 0 to 10 (like the *visual analogue scale*) [14] allowed this measurement to be easily and rapidly determined, and we wished to avoid using an anxiety-provoking terminology.

Comparison with international data was precluded by the absence of comparable studies in literature. Like others studies in primary care, results are depended on the health system organization. Comparisons must therefore be made within the same country. However, the issue addressed can be useful for all and shared in different countries.

Unfortunately, GP secretaries who systematically proposed parents with feverish children to participate in the study did not maintained a register of refusals. However, the number of inclusions per day in the study and the volume of consultations for fever in the region were superimposable, according to the data of the health monitoring services [27], thus limiting the risk of a large recruitment bias.

We did not ask for the diagnosis made by the doctor because the study was centered on the symptom “fever”. The short waiting period before the consultation, after the apparition of fever (1.3 ± 1.1 days), did not allow accurate diagnosis in all cases. The early treatment of disease at an undifferentiated (and therefore not accurately diagnosed) stage of development is a characteristic of general medicine [22]. A better knowledge of the populations treated in this context would aid the development of appropriate educational strategies.

## Conclusion

Family doctors in French rural areas reassure parents about their feverish child. Office opening hours should be organized to accommodate the reception of children with fever, and parents should be informed about the management of acute fever in their children.

We suggest that, despite an often-unfavorable medical demography in their areas, rural GPs organize their working conditions as those of the GP investigators of this study in order to facilitate access to consultations within a timeframe permitting to reduce parental concern.

## Additional file

Additional file 1: Parental questionnaire before and after GPs consultation. (DOCX 79 kb)

## Abbreviations

CS: Concern scale, concern score
GP(s): General practitioner(s)
ICPC-2: International classification of primary care, second edition
OR: Odd-ratio
RIAP: Individual activity and prescription record
