# Supplementary material for: Parental reassurance concerning a feverish child: determinant factors in rural general practice
Source: BMC Fam Pract. 2018 Jan 9;19:7. doi: 10.1186/s12875-017-0686-1 (PMC5759265; doi:10.1186/s12875-017-0686-1)
Supplement: Additional file 1: — Parental questionnaire before and after GPs consultation. (DOCX 79 kb) [file 12875_2017_686_MOESM1_ESM.docx]

Additional file 1

***STUDY “Parental reassurance concerning their feverish child: determinants in rural general practice.”***

***English language version (the French version was used with parents)***

QUESTIONNAIRE 1 – ***BEFORE*** GP consultation

Parental reassurance about acute fever in feverish children less than 6 years old.

**I- About you and your feverish child:**

1- Your child: age: … Sex: ☐M , ☐ F

2- Does your child have any medical records? ☐yes , ☐ no

If yes, which are they?: …

3- You are the child's: ☐ mother☐ father☐ other (specify)……

4- Your job: ….

5- Are you in a couple? ☐yes ,☐ no

6- How many siblings currently live at home?: ….

ages of each: ….

**II- About your child's fever**

7- Did you measure the child's temperature before your GP appointment? ☐yes ,☐ no

8- How long has you child been feverish? (specify days or hours):…

9- How would you define fever? Fever is a temperature of ….. °Celsius

10- How did you measure your child's temperature?

☐ with my hand ☐ I have no thermometer

☐ oral thermometer ☐ rectal thermometer

☐ armpit thermometer ☐ auricular thermometer

☐ frontal thermometer ☐ temporal thermometer

9- If measured, how much degrees did you add to the measured temperature? ….. °Celsius

11- According to you, fever is dangerous? ☐yes ,☐ no

If yes, starting from which temperature? ……°Celsius

12- According to you, what are the main complications of fever? …..

…..

13- On this scale from zero (no concern) to ten (maximal concern), how would you measure your concern about your feverish child **before** consultation?


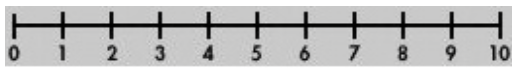


**III- Fever treatment:**

14- If your child has fever, how long do you usually wait until consulting your GP?

(specify days or hours) …….

15- If your child has fever:

- you undress your child : ☐ yes , ☐ no

- you give your child more water: ☐ yes , ☐ no

- you bathe your child: ☐ yes , ☐ no

- you ventilate his/her bedroom: ☐ yes , ☐ no

- you give medication: ☐ yes , ☐ no

16- Which medication would you firstly use to reduce fever? …..

- at which dose? ….

- how often? ….

- would you give other medications? if yes, specify which: ….

**IV- Information about fever:**

17-Which are your sources of information for managing your feverish child? (multiple choices are possible)

☐ my experience ☐ media (TV…)

☐ my general practitioner ☐ my entourage (family, friends…)

☐ my pediatrician ☐ my pharmacist

☐ my child’s health record

QUESTIONNAIRE 2 – Immediately ***AFTER*** GP consultation

1- On this scale from zero (no concern) to ten (maximal concern), how would you measure your concern about your feverish child **after** consultation?


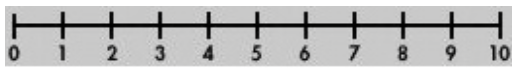


2- If you feel less concerned, why would that be?:

☐ GP gave a diagnosis

☐ GP examined my child

☐ GP questioned and listened to me

☐ GP gave us a prescription

☐ other (precise): ….

3- If you feel as concerned as before consultation, why would that be?: ….

4- If you feel more concerned, why would that be?: …
